# Free energy along drug-protein binding pathways interactively sampled in virtual reality

**DOI:** 10.1038/s41598-023-43523-x

**Published:** 2023-10-04

**Authors:** Helen M. Deeks, Kirill Zinovjev, Jonathan Barnoud, Adrian J. Mulholland, Marc W. van der Kamp, David R. Glowacki

**Affiliations:** 1 Center for Computational Chemistry, School of Chemistry, University of Bristol, Bristol, BS8 1TS UK; 2 Departamento de Química Física, Universidad de Valencia, 46100 Burjassot, Spain; 3 School of Biochemistry, University of Bristol, Bristol, BS8 1TD UK; 4 CiTIUS | Centro Singular de Investigación en Tecnoloxías Intelixentes da USC, Rúa de Jenaro de la Fuente, s/n, 15705 Santiago de Compostela, A Coruña Spain

**Keywords:** Chemistry, Theoretical chemistry, Computational biology and bioinformatics, Computational models

## Abstract

We describe a two-step approach for combining interactive molecular dynamics in virtual reality (iMD-VR) with free energy (FE) calculation to explore the dynamics of biological processes at the molecular level. We refer to this combined approach as iMD-VR-FE. Stage one involves using a state-of-the-art ‘human-in-the-loop’ iMD-VR framework to generate a diverse range of protein–ligand unbinding pathways, benefitting from the sophistication of human spatial and chemical intuition. Stage two involves using the iMD-VR-sampled pathways as initial guesses for defining a path-based reaction coordinate from which we can obtain a corresponding free energy profile using FE methods. To investigate the performance of the method, we apply iMD-VR-FE to investigate the unbinding of a benzamidine ligand from a trypsin protein. The binding free energy calculated using iMD-VR-FE is similar for each pathway, indicating internal consistency. Moreover, the resulting free energy profiles can distinguish energetic differences between pathways corresponding to various protein–ligand conformations (e.g., helping to identify pathways that are more favourable) and enable identification of metastable states along the pathways. The two-step iMD-VR-FE approach offers an intuitive way for researchers to test hypotheses for candidate pathways in biomolecular systems, quickly obtaining both qualitative and quantitative insight.

## Introduction

Recent advances in virtual reality (VR) technology have enabled new workflows across several scientific and engineering domains. For example, recent applications in nanoscience and microscopy use VR for interactively manipulating real-time dynamics of physical systems, guided by human scientific insight^1–3^. For understanding (bio)molecules, immersive technologies like VR have significant potential, given the fact that many molecular systems are characterized by considerable 3d complexity. The majority of work applying VR to molecular systems tends to focus on *interactive visualization*, e.g., Refs.^4–6^. Over the last few years, we have published a number of studies outlining strategies for *interactive simulation*, using an approach which we call interactive molecular dynamics in virtual reality (iMD-VR). Narupa, our open-source iMD-VR framework^7^, enables users to interact with a real-time molecular simulation as if it were a tangible dynamic object. Within this virtual environment, the player can reach out with a ‘force probe’ (i.e. a VR controller) and interactively manipulate the dynamics of molecular motion. In this way, a researcher can use their insight and expertise to guide molecules, using both spatial and chemical intuition to explore states of interest^8^.

The ability to manipulate molecules as if they were tangible objects is a unique means of studying molecular transformations, mechanisms, and rare events. Previous work has demonstrated that iMD-VR has acceleration benefits (2–10 ×) for performing 3*d* molecular tasks compared to 2*d* interfaces^7,9^. We have also shown iMD-VR to be a useful tool in building protein–ligand complexes^10^, with recent application to the SARS-CoV-2 main protease^11,12^. By coupling iMD-VR to quantum mechanical methods, we have also shown that reaction pathways can be efficiently sampled using iMD-VR^13^. When machine learning algorithms (e.g., atomic neural networks) are trained on these human-sampled reactive pathways, the learning rate is nearly 10 × faster than data sets obtained through more conventional brute-force sampling approaches^14,15^. Here, we show how iMD-VR can be used practically as a tool for enhancing the sampling of pathways through biomolecular conformational space, providing input for efficient free energy calculations that in turn provide the iMD-VR user with feedback.

Protein–ligand systems are high-dimensional and continually fluctuate between different conformations, often separated by kinetic or thermodynamic barriers. Such systems are increasingly being studied using molecular dynamics (MD) simulations, providing insight into their behaviour^16,17^. However, the dynamic and structural complexity of protein–ligand complexes makes them challenging to simulate. A single ligand unbinding event can take milliseconds, or even seconds, to occur^18,19^. While sampling of rare events in equilibrium simulations is computationally demanding and often not feasible, accelerated sampling techniques, such as umbrella sampling^20^ and metadynamics^21^, can be employed to reduce this load. However, many of these methods require defining a reaction coordinate (RC) along which to bias the simulation. A simple example of an RC would be to describe bond breaking as a single interatomic distance; here, the RC is made up of a single collective variable (CV), i.e., the bond distance. When applied to more complex molecular transitions however, basic RCs may not encode all the motions relevant to a molecular process (and therefore would not accurately control the progress of the transition). Defining an RC that uses more ‘collective variables’ (CVs) enables more sophisticated biasing, however, care still needs to be taken: A more detailed RC does not guarantee better guidance along the minimum free energy path of the transition (and may even be deleterious if CVs are included that are not relevant to the progress of the transition)^22^.

To describe complex molecular processes that can be understood as transitions between (meta)stable states, path collective variables (pathCVs) are one approach for defining RCs in high-dimensional space. Realistic trajectories of these processes (e.g. protein–ligand binding) are expected to be close to the minimum free energy path (MFEP) connecting those states. In such cases, a RC for the process can be defined as a path collective variable (pathCV) that changes smoothly when the system advances along the MFEP. Although a pathCV can be defined using MD snapshots along the MFEP, more sophisticated approaches will apply a transformation to this data, for example, by creating internal coordinates^23,24^, assigning different weights to the CVs in the distance calculation^25^, or employing the metric tensor defined by the geometry of the CV space^26^. Application of pathCVs in enhanced sampling can enable the sampling of complex processes, including enzyme-catalyzed reactions^27^ and large scale conformational changes in proteins^28^.

Sampling a process in a molecular system using pathCVs can be broken down into two stages: (i) discovering an (approximate) MFEP that connects two states and (ii) sampling along a pathCV defined using this MFEP. While the latter can be done with any enhanced sampling technique, it relies on being able to resolve a MFEP in the first place; a challenge in itself. Interactive molecular simulation environments (such as iMD-VR) can be used to take advantage of human spatial and chemical intuition of a complex conformational landscape. With careful direction using iMD-VR, a ligand can be placed into, or removed from, a protein binding pocket in 10–100 picoseconds of simulation time (taking only minutes of actual time)^10^. Tests of several protein–ligand systems showed that users (including non-specialists) can generate structures similar to those obtained by protein crystallography. Similarly, iMD-VR can be employed to generate candidate (un)binding pathways that explore an ensemble of conformations.

**Figure 1:**
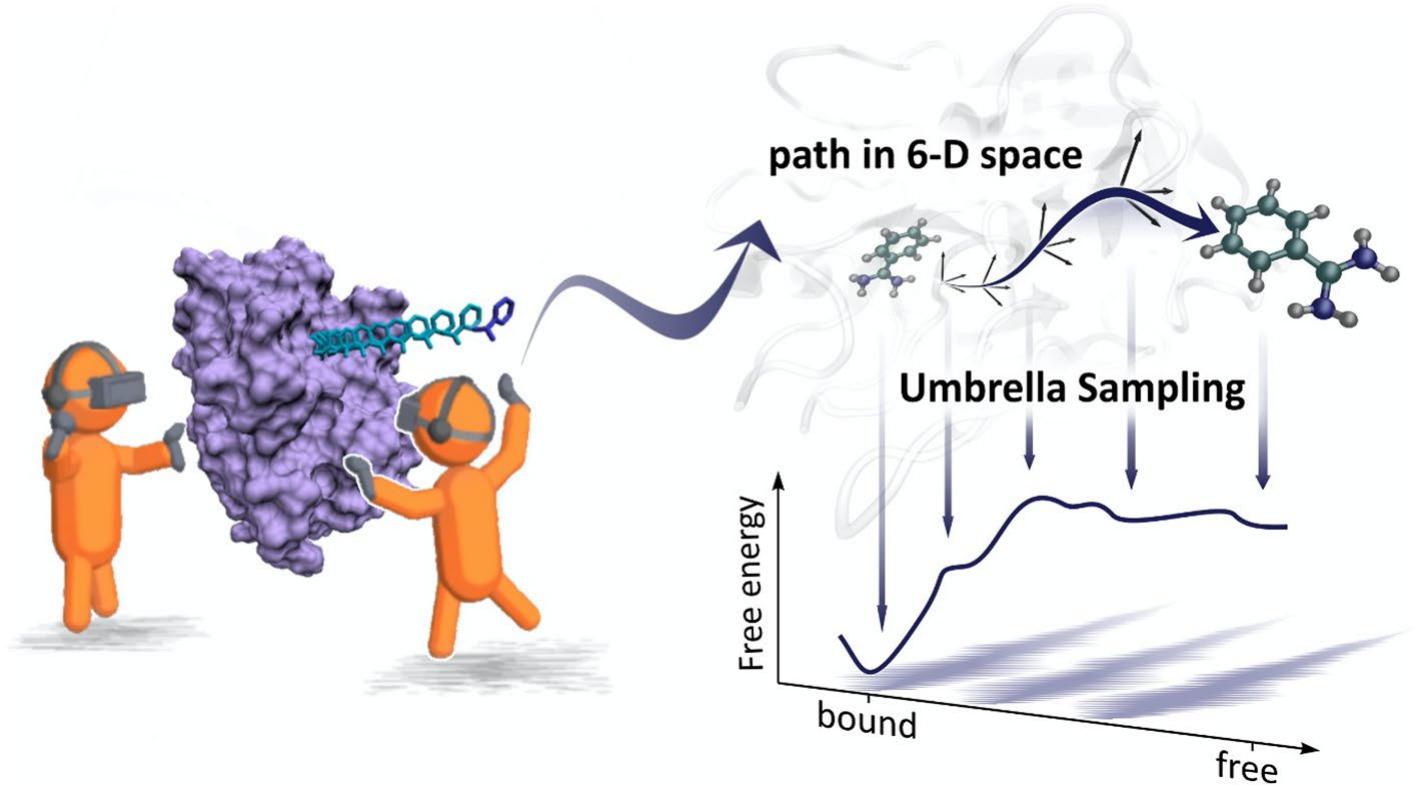
Workflow used to obtain free energy profiles for benzamidine unbinding from trypsin. First, users in iMD-VR model the dissociation of the ligand, by ‘pulling’ it out in an interactive MD simulation (left). Snapshots of the unbinding trajectory are used as input for umbrella sampling along a 6-D path collective variable (right) to obtain the free energy profile along the path.

In this work, we show how iMD-VR and free energy sampling techniques can be effectively combined to aid exploration of high-dimensional biomolecular systems, using as an example ligand dissociation from proteins. Figure 1 illustrates the workflow proposed here. Seven unbinding trajectories, or human-sampled paths, were generated within an iMD-VR simulation of the trypsin-benzamidine complex, a protein–ligand system with well characterized energetics^29–32^. Following Ref.^33^, we then projected snapshots along these paths into the space of six CVs that capture the position and orientation of benzamidine relative to trypsin. Using this reduced-dimensional descriptor, the free energies along these initial ‘guess’ pathways were calculated. We also explore how the adaptive string method^34^ can be used to gain quantitative suggestions for how inputs from iMD-VR can be optimized. The iMD-VR simulation presented here is available as a cloud-hosted iMD-VR service. Instructions on how to connect to the cloud simulation are given in the SI Appendix, alongside input files for running the iMD-VR simulation locally.

## Results

### Generation of iMD-VR pathways

**Figure 2:**
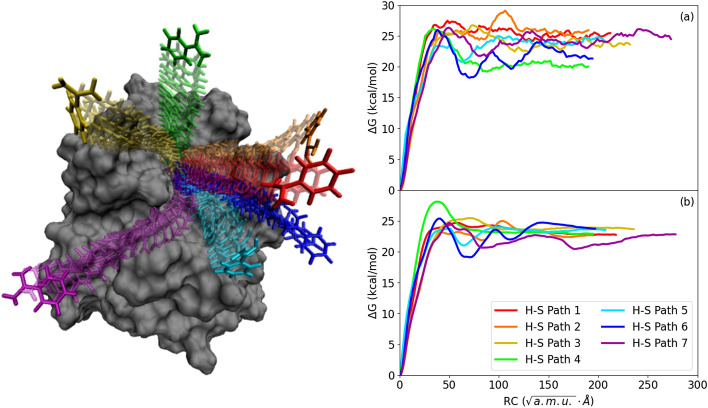
Benzamidine unbinding pathways and their free energy profiles. Left: The seven human-sampled benzamidine unbinding pathways obtained from iMD-VR superimposed onto the trypsin starting structure (based on PDB ID 1S0R). The color of each route corresponds to the line colors in the right-hand panel. Right: Free energy profiles calculated using the weighted histogram analysis method from 56 umbrella sampling windows along each of the 7 human-sampled paths. (**a**) Profiles obtained using only 10 ps of sampling in each US window; (**b**) profiles obtained using 1 ns of sampling per window.

Seven of the iMD-VR generated benzamidine unbinding pathways are shown in Fig. 2, each overlaid over a static representation of the trypsin surface. Starting from the bound complex, the user applied forces to the benzamidine ligand by selecting specific atoms and ‘pulling’ the controller in the desired direction, in order to move the ligand around relative to the protein. These forces are included alongside the regular force-field forces in the iMD simulation. Altogether, these human–sampled (H–S) paths were generated within an hour of laboratory time. Aside from an indication of the protein surface (through rendering the Van der Waals radius of each atom), the simulations did not include any specific visual guidance; the user simply aimed to move the ligand away from the protein. The substrate binding pocket (denoted as S1) sits buried in a larger groove on the protein surface. Restraints on the protein backbone were used to avoid large protein conformational changes (see “Methods”). Within the 3D iMD-VR environment, the researcher hypothesized multiple unbinding paths. H–S path 1 (red in Fig. 2) does not explore surface interactions; instead, benzamidine is pulled directly towards the bulk space. H–S paths 2–4 (orange, yellow, and green in Fig. 2) directed benzamidine away from the His57-Asp102-Ser195 catalytic triad and explored the steeper sides of the substrate pocket. In contrast, in H–S paths 5–7 (light blue, dark blue, and purple in Fig. 2) guided benzamidine was guided through the substrate binding pocket, moving it past the catalytic triad. Beyond that point, the binding pocket splits in two grooves extending in different directions. H–S path 5 did not explore this bifurcation and instead guided benzamidine straight into the bulk. However, for H–S paths 6 and 7, the researcher moved benzamidine down along one or other of these two grooves. iMD-VR provides a convenient approach to explore alternative pathways in 3D space^9^. Here, upon seeing a clear groove in the protein surface during the sampling of H–S path 7, the researcher decided to guide benzamidine close to the protein surface.

### Free energy sampling

Figure 2 shows the free energy along each of the seven iMD-VR trajectories (or H–S paths). The reaction coordinate was defined as a pathCV in a space of 6 CVs describing the ligand position and orientation relative to the protein (as proposed in Ref.^33^; see SI Appendix and Fig. S1 for details). Free energy profiles were obtained using umbrella sampling (US) MD simulations in two regimes: 2a shows the profiles integrated using only 10 ps of sampling in each of the 56 US windows, while 2b shows the profiles obtained from 1 ns sampling per window. The profiles from longer US are smooth, with differences in unbinding free energies within a few kcal/mol, indicating good convergence.

Not surprisingly, the free energy profiles obtained from short sampling (Fig. 2a) are much noisier and show significant variation of the estimated unbinding free energy. However, the most prominent features of the profiles obtained from longer sampling (approximate heights of the barriers and positions of the intermediate states) are already apparent. Thus, such ultra-short US provides quick estimate of the free energy along the ligand dissociation paths obtained from iMD-VR.

### Energetic characterization of iMD-VR generated pathways

The free energy of binding was estimated to be – 22.5 kcal/mol, larger than the experimental value of – 6.2 kcal/mol^35^. Our simulation protocols used a standard generalized Born (GB) implicit solvation model (that underestimates the benzamidine solvation energy), alongside positional restraints on the protein. Both can contribute to the significant overestimation of the binding energy. The positional restraints stabilize the bound state (present in the starting structure) and thus overestimate the energy of the unbound state (by 8–9 kcal/mole, Fig. S2). Binding energies calculated with GB implicit solvation often yield significantly different binding energies than the more accurate predictions with explicit solvent^36^. Overestimation of binding energies with the GB implicit solvent used here (OBC2) can be explained by the underestimation of small molecule solvation energies^37^. Taken together, a significant overestimation of the binding free energy was expected. Nonetheless, between paths, the estimated (un)binding free energy (the difference in energy between the starting complex and final unbound state) remained within a few kcal/mol, indicating that the obtained free energy profiles are well converged (with good convergence of the individual paths using 1 ns of sampling per US window; Fig. S3).

Most paths had a barrier height of approximately 25 kcal/mol corresponding to the iMD-VR user breaking the electrostatic contact with Asp189. However, H–S path 4 had a higher barrier as benzamidine was guided in a perpendicular direction to the S1 pocket opening, resulting in steric clashing against the roof of the pocket. H–S paths 1–4, which guided benzamidine away from the catalytic triad, generally had larger barrier heights. Of these four, path 2 guided benzamidine into a hydrophobic basin surrounding the S1 pocket, where the trypsin surface residues appear to adapt and form a cavity around the ligand. However, the user inadvertently guided the benzyl group against the polar Ser96 and Asp97 surface residues and caused a spike in free energy. The small minimum at a similar point for H–S path 6 is caused by the benzamidine being oriented such that the charged amidine group runs past these residues instead. Comparing the data in this work to the metastable states identified in Ref.^30^, H–S path 2 moved the ligand closest to state S1, H–S path 6 moved the ligand closest to state S3, and H–S path 7 moved the ligand closest to state S2.

**Figure 3:**
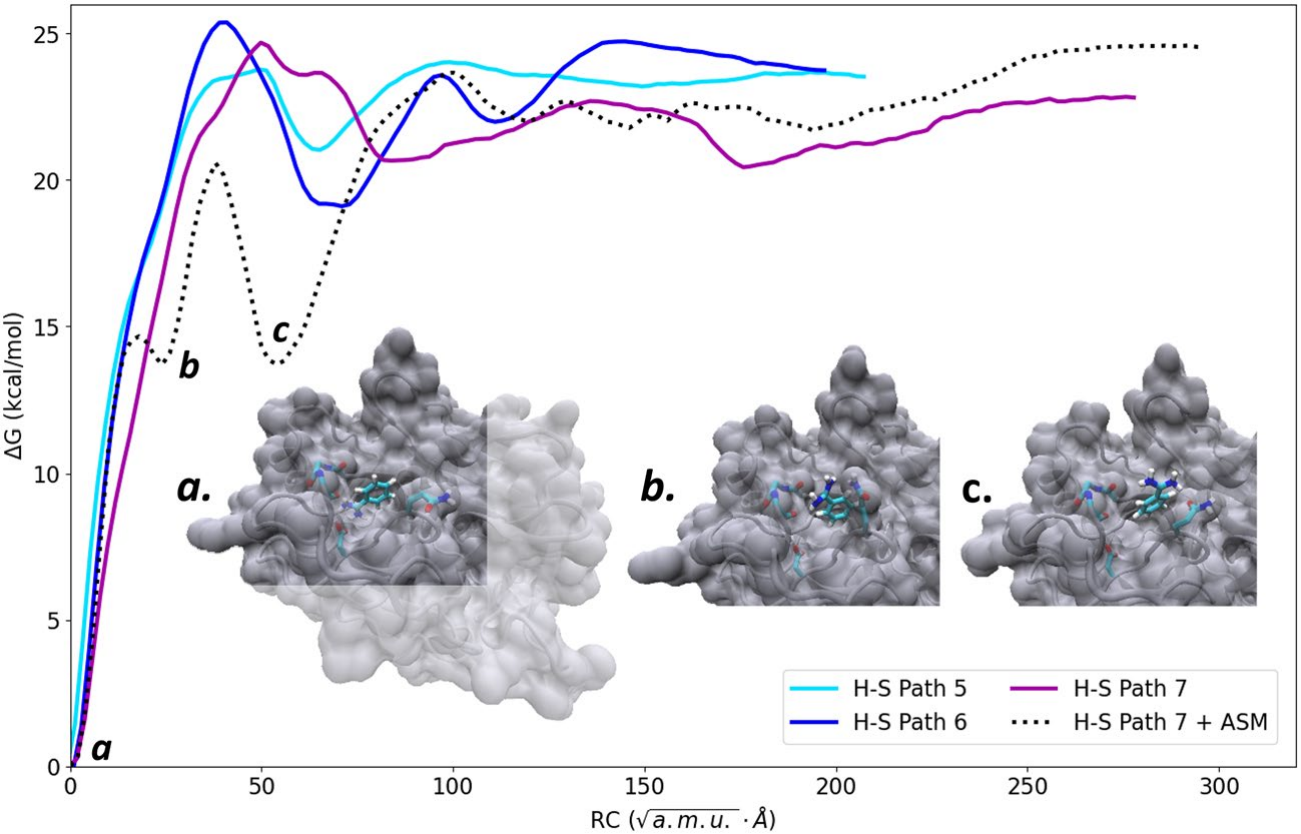
Free energy profile for path 7 refined using the adaptive string method (ASM). The dotted line and snapshots of key transitions depict the ASM-refined pathway, with the continuous lines for human-sampled paths 5–7 (from Fig. 2) shown for comparison. Representative snapshots from three points along the ASM-refined free energy profile are overlaid, showing how benzamidine rotates out of the binding pocket: (a) the starting bound state, (b) the first intermediate state, and (c) the second intermediate state.

Figure 3 shows the free energy profile of path 7 after refinement by ASM of the initial iMD-VR generated (H–S) path. There is a marked decrease in the free energy as benzamidine exits the S1 pocket. Specifically, two intermediate states are formed, corresponding to benzamidine rotating itself out of the S1 pocket. Figure 3a–c show representative snapshots of these states. In the first state, benzamidine rotates so that the contact with Asp189 is broken, but the hydrophobic group is buried in the space just outside the S1 pocket and favourable interactions are formed between the backbone oxygens of Gly214 and the Gln192 residue. In the second state, benzamidine has fully rotated itself such that the benzyl group is buried in the hydrophobic basin, specifically sandwiched between the alpha carbons of Cys191 and Trp211. The polar, charged amidine group is pointed towards the solvent and forms a closer interaction with Gln192. Notably, these two intermediate states are similar to states B and P described by Tiwary et al*.*, even though the reported water-mediated interactions are not captured due to our use of implicit solvent^38^. Nonetheless, the ASM refinement identifies new, stable states that are approximately 7 kcal/mol lower in energy than those sampled using iMD-VR alone, giving quantitative feedback on how future iMD-VR sampling could be improved (by guiding the ligand through such stable states). After these intermediate states, benzamidine remains close to the original iMD-VR path.

## Discussion

iMD-VR is an emerging tool for the quick exploration of complex molecular environments. Within a single hour-long laboratory session, iMD-VR was used here to generate seven unbinding pathways for benzamidine exiting the trypsin S1 pocket. Each H–S path served as a 'guess' for how unbinding could happen, driven by the iMD-VR user's own chemical and spatial intuition, alongside the forces from molecular dynamics. With reference to just a single distance, two angles and three dihedral angles from each molecular snapshot, any protein–ligand iMD-VR trajectory can be projected into a six-dimensional pathCV (see “Methods” and SI Appendix for details). As a result, inputs from iMD-VR can be used as the (initial) bias in enhanced sampling.

A carefully chosen RC can significantly reduce the computational resources required to simulate unbinding processes. However, selecting the CVs that capture the movement of a small ligand relative to a large, constantly fluctuating protein is a difficult challenge^22,39^. With increasing system complexity, hand selecting descriptors that are both comprehensive and high quality is impractical, especially where multiple pathways are being considered, as is often the case. Our aim was to perform a quick comparison of the free energy profiles related to the iMD-VR trajectories, and so we factored in how much sampling was needed for convergence. While a simple RC (such as distance between the ligand and some group in the active site) would be fast to implement, it would not contain any information about the direction and rotation of the ligand. Therefore, there is little guarantee that the sampling would follow the iMD-VR trajectory. On the other hand, an arbitrarily complex RC will probably better follow the iMD-VR paths, but may also include redundant information (such as conformational changes in the protein or ligand unrelated to the path, sampled accidentally in iMD-VR) without adding much value. We identified a set of six positional and rotational descriptors (see SI Appendix) as the minimal amount of information needed to unambiguously identify the ligand position and orientation with respect to the protein. This approach is particularly suitable for representing iMD-VR pathways because these descriptors can be measured during the interactive simulations, immediately projecting the user-sampled pathway onto a reduced dimensional space.

We demonstrate that 10 ps US per window is sufficient to provide a reasonable approximation of the underlying free energy profile (Fig. 2). The protocol demonstrated here can be expanded to ‘on-the-fly' integration with VR, which could eliminate a posteriori analysis of the full iMD-VR pathways and help guide the user. For example, H–S path 2 had an initially promising free energy profile, up until the user accidentally clashed the ligand against a hydrophobic surface residue. This makes H–S path 2 is of limited value, although, with a small adjustment, the user could instead explore a surface groove near this residue (which would lead the system towards a previously observed metastable state)^30^, leading to a lower barrier. The feasibility of such an approach will depend on the size of the system, available hardware and efficiency of the simulation software used. For moderately large protein–ligand complexes, modern high-end GPUs can provide up to microseconds of MD sampling per day for systems of this size^40,41^, or approximately 10 ps of sampling in 1 s of GPU time. Therefore, an on-the-fly adaptation of this protocol is theoretically within reach.

To minimize computational load and simplify the experimental pipeline, our simulation protocol included some approximations. The iMD-VR simulations here employed implicit solvation, and so for consistency between iMD-VR and US, the same approach was applied throughout. Water molecules are thought to play an important role in benzamidine binding to trypsin^42^, including water-mediated stabilizing interactions for intermediate states^38^, so lack of explicit solvent is not ideal. We also employed protein backbone restraints to limit the conformational space accessible during iMD-VR, to avoid large changes due to the high forces applied. Additional benefits of this are that consistency between H–S paths is improved, because the global protein structure is prevented from diverging between paths, and it allows for faster convergence for both US and path optimization with the ASM. As the protein movement is restricted, some resolution of the unbinding process may be lost. For example, just before exiting the binding pocket in H–S path 7, a favourable interaction is briefly formed with Tyr39. However, a previously suggested metastable state has benzamidine sandwiched between this residue and Tyr151^30^. As the protein had a limited range of movement in our simulations, benzamidine could not contact both tyrosine residues simultaneously during sampling. Backbone restraints are also likely to artificially increase free energy differences between bound and unbound states: there is a bias towards the protein conformation in the starting, bound complex, which in turn causes US to overestimate its stability.

Nonetheless, given that iMD-VR trajectories can include energetic artifacts due to the bias applied by the user (such as the energy spike seen in H–S path 2), there is limited benefit to using an expensive sampling protocol as a first pass. We further do not recommend that calculated free energies from our suggested protocol are treated as accurate. Instead, this protocol should be used to evaluate iMD-VR ‘guessed’ paths relative to one another. Here, it was found to be more favourable to direct benzamidine through a large groove on the trypsin surface and towards the catalytic triad, especially in the direction of metastable states identified in other work^30^. Refinement using the ASM method can further be used to quantify where iMD-VR generated paths can be improved. Here, the user guided benzamidine from the S1 pocket benzyl group first, resulting in a sharp energy barrier. With ASM refinement, however, the trajectory samples benzamidine rotating out of the S1 pocket, as seen in other work^30,38^. This gives feedback to the iMD-VR user that they should not pull benzamidine out with the hydrophobic ring directly pointing at the solvent. Such changes towards a more energetically feasible pathway could also be obtained by using ‘on-the-fly’ integration of iMD-VR with pathCV-based enhanced sampling. Promising pathways could be repeatedly sampled in iMD-VR, whilst optimizing them to reach low energy barriers and metastable states. We propose the following software pipeline: (i) The user generates a trajectory of an unbinding pathway using iMD-VR; (ii) individual snapshots are projected onto 6d-space as soon as they are generated; (iii) the new points are added to the pathCV definition and an additional US window is defined and sampled; (iv) free energy data for the unbinding path up to the new point is passed back to the user. We anticipate this ‘on-the-fly’ feedback would prompt the user to explore more favourable regions of conformational space, making the iMD-VR session more productive. Additionally, only the more promising paths would be chosen for more extensive sampling and detailed analysis, reducing computational load.

The trypsin-benzamidine system is a good proof-of-principle for our workflow, because it is well understood (through extensive simulation with more conventional approaches). The version of pathCVs used here^26^ can characterize a large number of interdependent coordinates in an arbitrary number of dimensions. Therefore, by also including internal coordinates from the protein, this workflow could be used for other protein–ligand systems (e.g. where the motion of a lid-like domain often controls ligand release). While it is unlikely that a single researcher generating a single unbinding pathway will perfectly characterize a MFEP, it is apparent that iMD-VR can be used to quickly sample a range of physically reasonable pathways. It is possible to run iMD-VR remotely, hosted on the cloud, which would allow scientists (and non-scientists) from around the world to be recruited to generate a large ensemble of paths. Generated pathways can be 'scored' by iMD-VR-FE, and hence this problem gamified, with researchers aiming to find paths with low barriers (and metastable states)^13^. These data, leveraging human intuition, can then guide accelerated sampling methods along these paths, allowing intelligent, human-led exploration of complex dynamics. In understanding the relative energetics of a bound state, its surrounding metastable regions and feasible unbinding pathways, users can gain insights that aid drug design. This complements other, non-interactive methods for binding pathway exploration^43,44^﻿ as well as more computationally intensive, non-interactive enhanced sampling approaches^29–32,38,42,45,46^. Given that iMD-VR can also be used to create protein–ligand complexes for which an experimental structure does not exist^11^, the iMD-VR-FE protocol may be especially suited towards areas such as discovery and development of allosteric drugs. Here, we present an initial implementation of this protocol; we anticipate that it will be developed further for multiple different applications.

## Methods

### iMD-VR for sampling of protein–ligand unbinding pathways

#### System setup

Trypsin was parameterized with the Amber ff14SB forcefield^47^, benzamidine was parameterized with the general amber forcefield (GAFF) and AM1-BCC charges^48^ in Antechamber^49^, and the solvent was modelled implicitly using the OBC2 generalized Born model.^50^ Prior to using iMD-VR to generate unbinding pathways, the complexed structure, with starting coordinates from PDB ID 1S0R, was minimized and equilibrated. The details of this process are given in Section 2 of the SI Appendix.

#### iMD-VR simulations

A minimized and equilibrated complex of benzamidine bound to the S1 pocket of trypsin was used as the starting point for iMD-VR. An iMD-VR proficient user then proceeded to carefully guide the ligand out of the binding pocket. Harmonic positional restraints were used for the protein backbone atoms CA, N, O, C (10 kcal mol^−1^ Å^−2^) and the Ca^2+^ ion (20 kcal mol^−1^ Å^−2^). A total of seven different iMD-VR unbinding pathways were generated, each taking a distinct route (shown in Fig. 2). With the provided Narupa simulation files, researchers can set up their own locally hosted simulation environments. We also make a Narupa iMD-VR demo of the trypsin-benzamidine interactive simulations available via cloud infrastructure, which can be launched from app.narupa.xyz. Instructions for connecting to this demo can be found in Section 1 of the SI Appendix.

### Calculating free energies along protein–ligand unbinding pathways

#### Definition of the pathCVs

The unbinding pathways obtained from iMD-VR were first characterized by 6 simple CVs describing relative orientation of the two species based on 3 reference points on each, as proposed in^33^ (Fig. S1). These reference points for each species (protein and ligand) were chosen such that their geometric centers form approximately equilateral triangles and their positions are not easily affected through thermal fluctuations. Explicit definitions of the CVs are included in Section 3 of the SI Appendix.

After the pathways were projected onto the selected CV space, the pathCVs were used to: (a) define a reaction coordinate (RC) that changes smoothly along the path, and (b) ensure that the simulation system stays in the vicinity of the path. The metric-corrected^26^ version of the pathCVs was used to account for different functional forms and couplings (distance, angles and dihedral angles) of the chosen CVs. Definition of the pathCVs is provided in the SI Appendix (Section 3).

#### Free-energy calculation

The free energy profiles along the pathCVs were calculated using US^20^. This consists of running a set of simulations biased to different values of the chosen RC with harmonic biasing potentials and subsequent integration of the obtained sampling to recover the full free energy profile. The same setup and simulation protocol were used for all 7 H–S paths. Details of the protocol can be found in Section 4 of the SI Appendix. Briefly, all the US simulations were performed with a modified version of sander from AmberTools (https://github.com/kzinovjev/string-amber) using the same parameterization and implicit solvent model as used in iMD-VR. 56 US windows were used. The initial structures for US windows were obtained by taking the closest snapshot from the VR pathway and running 1 ps MD, while gradually increasing the force constant from zero to the target value. 1 ns of sampling was acquired for each window during production simulations. The resulting potentials of mean force were integrated using the weighted histogram analysis method (WHAM)^51^. The additional analysis carried out on path 7 (Fig. 3) utilized the adaptive string method^34^. Path optimization was performed using an extension of the sander code, which is available on GitHub (https://github.com/kzinovjev/string-amber). Details of the optimization protocol can be found in Section 5 of the SI Appendix.

### Supplementary Information

Supplementary Information.

## Data Availability

The programs necessary for running a standalone simulation of trypsin and benzamidine in Narupa are available at: https://gitlab.com/intangiblerealities/narupa-protocol. Simulation parameters, input files for Narupa, the seven iMD-VR guided trajectories, reference pathways and the free energy profiles for all the runs are available via https://doi.org/10.5281/zenodo.6659616, as additional Supporting Information.
