# Supplementary material for: Free energy along drug-protein binding pathways interactively sampled in virtual reality
Source: Sci Rep. 2023 Oct 4;13:16665. doi: 10.1038/s41598-023-43523-x (PMC10551034; doi:10.1038/s41598-023-43523-x)
Supplement: Supplementary file 1 — Supplementary Information. [file 41598_2023_43523_MOESM1_ESM.pdf]

Free energy along drug-protein binding pathways interactively sampled in virtual reality

Supporting Information

Helen M. Deeks,[1,†] Kirill Zinovjev,[2,3†] Jonathan Barnoud[1,4], Adrian J. Mulholland[1], Marc W. van der Kamp[1,3*], David R. Glowacki[,4*]

[†]*These authors contributed equally this work*

[1]*Center for Computational Chemistry, School of Chemistry, University of Bristol, Bristol, BS8 1TS, UK,* [2]*Departamento de Química Física, Universidad de Valencia, Burjassot, 46100, Spain,* [3]*School of Biochemistry, University of Bristol, Bristol, BS8 1TD, UK,* [4]*CiTIUS Intelligent Technologies Research Centre, Rúa de Jenaro de la Fuente, s/n, 15705 Santiago de Compostela, A Coruña, Spain*

**marc.vanderkamp@bristol.ac.uk; *drglowacki@gmail.com*

1. Connecting to a cloud-hosted iMD-VR simulation

1.1 Requirements

The VR client has been developed on Microsoft Windows using the HTC Vive Pro, the Valve Index, the Oculus Rift, the Oculus Rift S, and both the Oculus Quest and Quest 2 using Oculus link. In all cases, a VR-capable computer running both Windows 10 and SteamVR is necessary. The server runs remotely and data is sent to the VR client over the internet. Therefore, a stable internet connection is required. For a simulation size comparable to trypsin-benzamidine, a 10 Mbps download speed is the recommended minimum.

1.2 Run a server in the cloud

A Narupa server runs the molecular dynamics simulation, sends the frames to the clients, and synchronises the positions of the user's avatars. The Narupa Cloud interface can be accessed at https://app.narupa.xyz. Upon first use of the Narupa Cloud service, a user needs to create an account. Only one user needs to start a server instance; once the server is running, other users can connect to it without an account on the service.

Once logged into Narupa Cloud, go to the "Sessions" section of the site. The page lists the current and future server sessions that are scheduled by the user. By clicking on the "Schedule a session" button, a user can start a server session now, or schedule one in the future.

On the scheduling page, select the simulation named "Trypsin-Benzamidine", the starting time and the duration you want. Ideally, you would select the server closest to your location (this improves latency). Clicking the "Schedule" button at the bottom of the page will validate your choices and submit the scheduling request. The scheduled session should then appear in the list on the "Sessions" page. Server sessions scheduled to start immediately needs a few minutes to be ready; session scheduled for the future should be ready at the requested time. When the session is ready, an IP address appears next to the session on the "Sessions" page. This IP address is needed for the VR clients to connect and should be sent to all intended users.

1.3 Connect a VR client

Before running the VR client, make sure the VR headset is plugged and ready to go. You need SteamVR installed. More information on SteamVR can be found at (https://store.steampowered.com/app/250820/SteamVR)

Each user needs to download the latest version of Narupa iMD at (https://gitlab.com/intangiblerealities/narupa-applications/narupa-imd/-/jobs/artifacts/master/download?job=build-StandaloneWindows64). The file behind that link is named "artifact.zip", decompress it at the location of your choice. In the resulting directory, move to the "Builds" directory, then the "StandaloneWindows64" one, and double click on the "Narupa iMD.exe" file. Windows will display a security warning because it does not know about Narupa, click on "More info", then "Run anyway". At this point, Narupa iMD should run and should be displayed both on screen and in the VR headset. On the top left of

the computer screen, click on “Direct connect”, set the address to the IP address of your server session and click “Connect”.

1.4 Adjust the visual representations

Changing how the molecules are represented in VR requires to connect to the server session with a python client. To install the python client of Narupa, follow the instructions at (https://gitlab.com/intangiblerealities/narupa-protocol#quick-installation-for-a-user)

You will also need to install jupyter to run the provided notebook. In the Anaconda Powershell Prompt used in the previous steps, execute the following commands:

```
conda activate Narupa
conda install jupyter
jupyter notebook /the/path/to/the/downloaded/notebook
```

An example visual representation notebook can be found in the ‘simulations’ folder of the supplementary materials. Change the IP address at the top of the notebook to the one of the server session and run all the cells.

2. Trypsin-benzamidine system set-up

Hydrogen atoms were added to the protein and ligand using reduce. The protein structure was parameterized with the Amber 14 force field (1) and benzamidine was parameterized with the General Amber Forcefield (2) in antechamber, using AM1-BCC partial charges. The solvent in each simulation was modelled implicitly using OBC2. (3)

Prior to simulation in iMD-VR, the structure was minimized and equilibrated in implicit solvent. First, the structure was iteratively energy minimized using slowly decreasing degrees of positional restraint. 5 kcal/mol/A^2, 2.5 kcal/mol/A^2, and 1.25 kcal/mol/A^2 was applied to all backbone and ligand atoms for the first three rounds of minimization respectively, and no restraints were applied for the final round. Next, the system was heated by running 10 stages total of 20 ps of molecular dynamics, starting at 0K and linearly increasing the temperature by 30K at each stage until a temperature of 298K was reached (each step had a backbone and ligand atom restraint of 5 kcal/mol/A^2).

Finally, 8 rounds of 500 ps of molecular dynamics with slowly decreasing backbone and ligand atom restraints was run to equilibrate the structure. Restraints were initially 5 kcal/mol/$Å^2$ and halved after each step; once backbone restraints were below 1 kcal/mol/A^2, the restraint atoms were reduced to only C-alpha backbone atoms and ligand atoms. The eighth and final stage had no restraints on the protein and ligand at all. All stages of minimization had a 20 kcal/mol/ A^2 positional restraints on the calcium ion embedded within the trypsin structure.

Due to the introduction of artificially high forces during iMD-VR, a strong backbone positional restraint was applied to the trypsin structure and single calcium ion during interactive simulations. All other parameters were taken directly from the minimization and test production MD. For all iMD-VR simulations, a temperature of 300K was used with a timestep of 0.5 fs. Snapshots of the iMD-VR simulations were taken every 500 timesteps, equal to every 0.25 ps.

3. Definition of the reaction coordinate

The unbinding pathways obtained from VR were characterized by 6 collective variables (CVs) describing relative orientation of the two species as proposed in (4):

$$r = distance(P_1L_1);\ \theta = angle(P_1L_1L_2);\ \phi = dihedral(P_1L_1L_2L_3)$$

$$\Theta = angle(\mathrm{P}_2\mathrm{P}_1L_1);\ \Phi = dihedral(P_2P_1L_1L_2);\ \Psi = dihedral(P_3P_2P_1L_1)$$

Where P1-P3 and L1-L3 are reference centers in the protein and the ligand respectively, defined as geometric centers of the following atom groups:

**Protein centers (all non-hydrogen atoms of following residues)**	**Ligand centers (atoms)**
P1: Cys136, Leu137, Ile138, Cys157, Leu158, Lys159, Pro198, Val199, Val200	L1: C, C1, N1, N2
P2: Phe181, Cys182, Ala183, Val213, Ser214, Trp215, Gly226, Val227, Tyr228	L2: C2, C3
P3: Val31, Ser32, Leu33, Phe41, Cys42, Gly43, Gln64, Val65, Arg66	L3: C5, C6

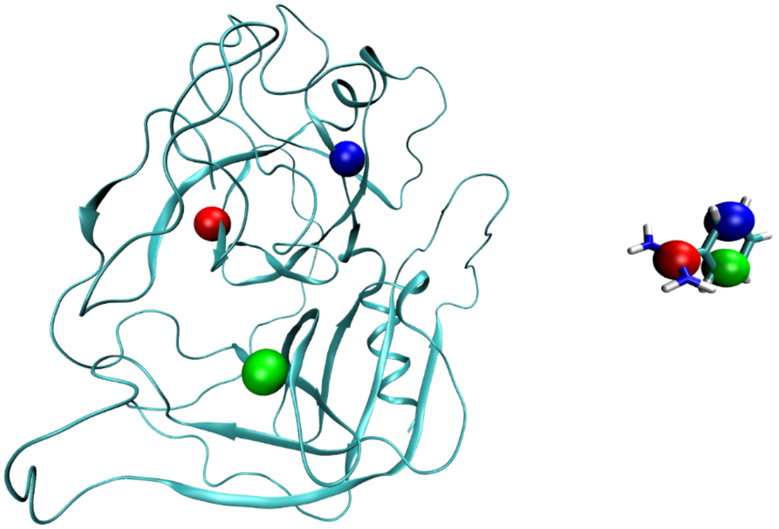

Figure S1: Geometric centers used in CV definition. P1/L1, P2/L2/ and P3/L3 are represented with red, green and blue spheres respectively.

These reference points for each species (protein and ligand) were chosen such that their geometric centers form approximately equilateral triangles that are not sensitive to thermal fluctuations, which in case of the protein means avoiding the use of residues present in flexible loops. By choosing this representation we assume that the internal degrees of freedom (such as vibrations and conformational changes) are not coupled to the unbinding and therefore can be excluded from the reaction coordinate. While this assumption is valid for the trypsin-benzamidine case, it would fail if there were a significant conformational rearrangement during the unbinding (e.g. lid opening-like motion). In such case, an additional CV describing the conformational change must be included.

To obtain the free energy profiles along iMD-VR pathways one needs to 1) define a reaction coordinate (RC) that changes smoothly along the path and 2) ensure that the simulation system stays in the vicinity of the path. The latter is especially important when the user intentionally generates a sub-optimal path to explore an alternative unbinding mechanism. Without any additional restraint, there is a high chance of the system reverting to a more favorable path (which might have already been explored). Both tasks can be accomplished with path collective variables (pathCVs) (5) defined along the paths in the chosen CV space (6):

$$s(\boldsymbol{r}) = \frac{\sum_{i=1}^{n} t_i e^{-\lambda d(\boldsymbol{\theta}(\boldsymbol{r}),\boldsymbol{z}(t_i))}}{\sum_{i=1}^{n} e^{-\lambda d(\boldsymbol{\theta}(\boldsymbol{r}),\boldsymbol{z}(t_i))}}; \qquad z(\boldsymbol{r}) = -\lambda^{-1} \ln \sum_{i=1}^{n} e^{-\lambda d(\boldsymbol{\theta}(\boldsymbol{r}),\boldsymbol{z}(t_i))}; \qquad t_i = \frac{i-1}{n-1} L$$

Where $\boldsymbol{z}(t)$ is the reference path parameterized by the arc length, L is the total length of the path and $d(\boldsymbol{\theta}(\boldsymbol{r}), \boldsymbol{z})$ is some distance measure between the given state of the system $\boldsymbol{r}$ and a point $\boldsymbol{z}$ on the reference path and $\boldsymbol{\theta}(\boldsymbol{r})$ is the 6-vector of CVs defined above. λ is set to inverse of the distance between the points $\boldsymbol{z}(t_i)$. To account for interdependency and different nature of the CVs, the following measure was used (7):

$$d(\boldsymbol{\theta}(\boldsymbol{r}), \boldsymbol{z}(t_i)) = \sqrt{\left(\boldsymbol{\theta}(\boldsymbol{r}) - \boldsymbol{z}(t_i)\right)^T \boldsymbol{M}(t_i)^{-1} \left(\boldsymbol{\theta}(\boldsymbol{r}) - \boldsymbol{z}(t_i)\right)}$$

Where $\boldsymbol{M}$ is the variable distance metric tensor defined as:

$$\boldsymbol{M}(t_i)_{jk} = \langle \nabla \theta_{\mathrm{j}}(\boldsymbol{r}) \cdot \nabla \theta_{\mathrm{k}}(\boldsymbol{r}) \rangle_{\boldsymbol{\theta}(\boldsymbol{r}) = \boldsymbol{z}(t_i)}$$

Where gradients are taken with regards to mass-weighted Cartesian coordinates and $\langle \dots \rangle_{\boldsymbol{\theta}(\boldsymbol{r}) = \boldsymbol{z}(t_i)}$ denotes canonical ensemble average over configurations constrained to the point $\boldsymbol{z}(t_i)$ in the CV space.

4. Umbrella sampling protocols

The same setup and simulation protocol were used for all 7 human-sampled paths. All the simulations were performed with a modified version of sander from AmberTools19 (https://ambermd.org/CiteAmber.php). Generalized Born implicit solvation method was used to describe the water solution. Temperature was set to 300K and was controlled with Langevin thermostat. The bonds involving hydrogen were constrained using SHAKE, which allowed to set the integration timestep to 2fs. 56 Umbrella Sampling windows were used. Harmonic biases were equally spaced along the range of pathCV values with force constants determined automatically to guarantee uniform sampling assuming flat underlying free energy profile (see (8) for details). The initial structures for US windows were obtained by taking

the closest snapshot from the VR pathway and running 1 ps MD gradually increasing the force constant from 0 to the target value. 1 ns of sampling was acquired during production simulations. Hamiltonian replica exchange between windows was attempted every 500 fs. To restrain the sampling to the vicinity of the path, a harmonic bias was added along the z coordinate at $z = 0$ with force constant = 1 $kcal \cdot mol^{-1} \cdot a.m.u.^{-1} \cdot Å^{-2}$. The resulting potentials of mean force were integrated using WHAM procedure. (9)

5. String method protocols

The adaptive version (8) of on-the-fly string method (10) was used to obtain the minimum free energy path in the vicinity of path 7. The following ASM parameters were changed from their default values to ensure faster convergence and stability of the simulations: $\gamma = 500\, ps^{-1}, K^{\perp} = 1\, kcal \cdot mol^{-1} \cdot a.m.u.^{-1} \cdot Å^{-2}, \gamma_{\alpha} = 200\, kcal \cdot mol^{-1} \cdot a.m.u.^{-1} \cdot Å^{-2} \cdot ps, \kappa = 1\, a.m.u.^{-2} \cdot Å^{-4}$. Same CVs as for the pathCV Umbrella Sampling calculations were used in the string method. Path 7 was used as the initial guess. Endpoints of the path were fixed in the CV space. The MD protocol was identical to the one used for Umbrella Sampling. The string optimization converged after 1.7 ns of simulation. The free energy along the converged path was obtained using the same procedure as for the paths obtained directly from VR.

6. Restraint and convergence tests

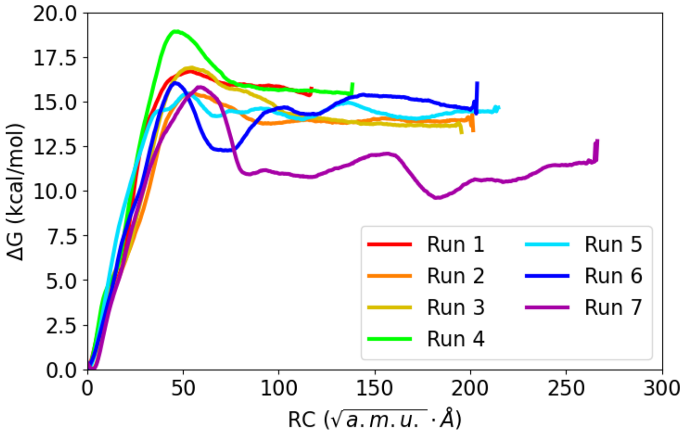

Figure S2: Effect of positional restraints on protein atoms. Free energy profiles calculated using the weighted histogram analysis method along the 7 human-sampled paths, without positional restraints on protein atoms.

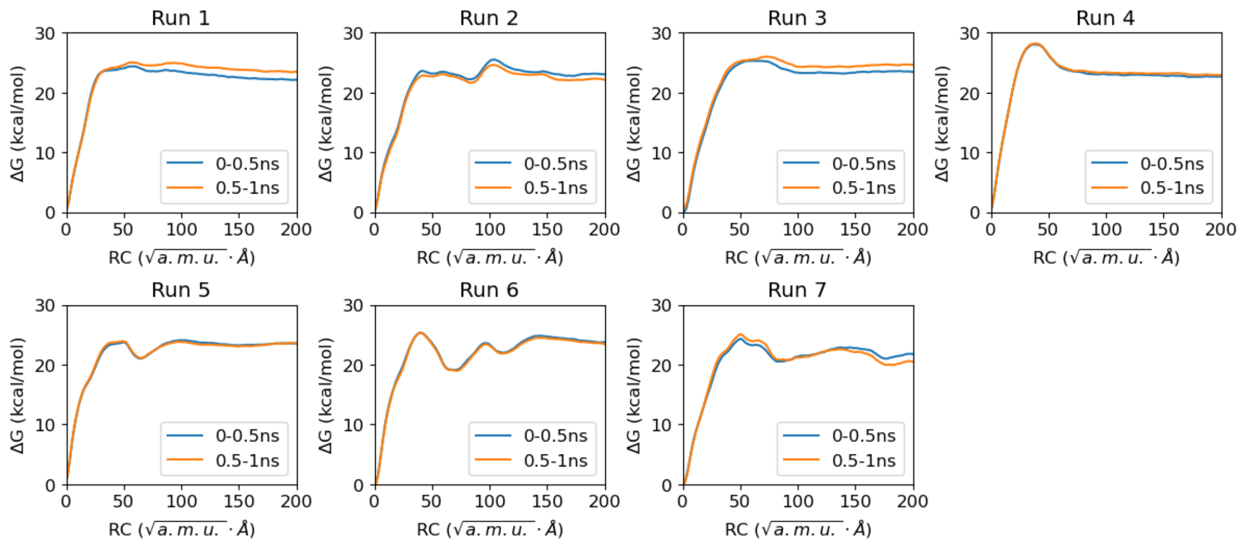

Figure S3: Analysis of convergence of the sampled free energy profiles. For the free energy profiles obtained with 1 ns of sampling per window, the profiles obtained with the first and last 0.5 ns of sampling are shown, suggesting that further sampling would not lead to significant differences.

References

1. Maier JA, Martinez C, Kasavajhala K, Wickstrom L, Hauser KE, Simmerling C. ff14SB: Improving the Accuracy of Protein Side Chain and Backbone Parameters from ff99SB. J Chem Theory Comput. 2015;11(8):3696-713.
2. Wang J, Wolf RM, Caldwell JW, Kollman PA, Case DA. Development and testing of a general amber force field. J Comput Chem. 2004;25(9):1157-74.
3. Onufriev A, Bashford D, Case DA. Exploring protein native states and large-scale conformational changes with a modified generalized born model. Proteins: Structure, Function, and Bioinformatics. 2004;55(2):383-94.
4. Suh D, Jo S, Jiang W, Chipot C, Roux B. String Method for Protein-Protein Binding Free-Energy Calculations. J Chem Theory Comput. 2019;15(11):5829-44.
5. Branduardi D, Gervasio FL, Parrinello M. From A to B in free energy space. J Chem Phys. 2007;126(5):054103.
6. Zinovjev K, Marti S, Tunon I. A Collective Coordinate to Obtain Free Energy Profiles for Complex Reactions in Condensed Phases. J Chem Theory Comput. 2012;8(5):1795-801.
7. Zinovjev K, Tunon I. Exploring chemical reactivity of complex systems with path-based coordinates: role of the distance metric. J Comput Chem. 2014;35(23):1672-81.

8. Zinovjev K, Tuñón I. Adaptive Finite Temperature String Method in Collective Variables. The Journal of Physical Chemistry A. 2017;121(51):9764-72.
9. Kumar S, Rosenberg JM, Bouzida D, Swendsen RH, Kollman PA. THE weighted histogram analysis method for free-energy calculations on biomolecules. I. The method. Journal of Computational Chemistry. 1992;13(8):1011-21.
10. Maragliano L, Vanden-Eijnden E. On-the-fly string method for minimum free energy paths calculation. Chemical Physics Letters. 2007;446(1):182-90.
